# Supplementary material for: Impact of axisymmetric deformation on MR elastography of a nonlinear tissue-mimicking material and implications in peri-tumour stiffness quantification
Source: PLoS One. 2021 Jul 9;16(7):e0253804. doi: 10.1371/journal.pone.0253804 (PMC8270167; doi:10.1371/journal.pone.0253804)
Supplement: S1 Appendix — (ZIP) [file pone.0253804.s001.zip › S1 appendix.pdf]

Supporting information

S1 Appendix. Linearised elastic wave equations in the presence of a macroscopic deformation.

The Cauchy momentum equation and the continuity equation describe the finite-deformation mechanics of a body. Their incompressible Lagrangian forms read

$$\rho J \frac{\partial^2 \boldsymbol{U}}{\partial t^2} - \nabla_{\boldsymbol{X}} \cdot \boldsymbol{P} = 0 \qquad \Omega_0 \tag{1a}$$

$$J - 1 = 0 \qquad \Omega_0 \tag{1b}$$

Considering the low-amplitude harmonic waves employed in MRE as a perturbation of the underlying macro-deformation, the two deformation fields and their associated hydrostatic pressures can then be linearly combined as follows:

$$\boldsymbol{U}^{\varepsilon} = \boldsymbol{U} + \boldsymbol{u}_{\varepsilon} \tag{2a}$$

$$P^{\varepsilon} = P + p_{\varepsilon} \tag{2b}$$

where

$$\boldsymbol{u}_{\varepsilon}(\boldsymbol{x}, t) = \mathrm{Re}\{\boldsymbol{u}_{\mathbb{C}}(\boldsymbol{x}) e^{i\omega t}\} \qquad \text{and} \qquad p_{\varepsilon}(\boldsymbol{x}, t) = \mathrm{Re}\{p_{\mathbb{C}}(\boldsymbol{x}) e^{i\omega t}\} \tag{3}$$

are complex-valued functions of space. Given the scale separation, a new perturbed version of the equations of motion can be defined:

$$\rho J^{\varepsilon} \frac{\partial^2 \boldsymbol{U}^{\varepsilon}}{\partial t^2} - \nabla_{\boldsymbol{X}} \cdot \boldsymbol{P}^{\varepsilon} = 0 \qquad \Omega_0 \tag{4a}$$

$$J^{\varepsilon} - 1 = 0 \qquad \Omega_0 \tag{4b}$$

where the superscript ε indicates the quantities dependent on the perturbed state variables. These equations can be simplified through the linearisation of the expansion around $\boldsymbol{U}$ with respect to the perturbations $\boldsymbol{u}_{\varepsilon}$ and p_{ε}. To do so, we followed the steps proposed by [1]. The full derivations of the linearised PK1 stress tensor has been produced using [2]. By explicitly writing the dependence of the PK1 tensor on the perturbed macro-deformation $\boldsymbol{U}^{\varepsilon} = \boldsymbol{U} + \boldsymbol{u}_{\varepsilon}$ and pressure $P^{\varepsilon} = P + p_{\varepsilon}$

$$\boldsymbol{P} = \boldsymbol{P}\left(\boldsymbol{F}(\boldsymbol{U} + \boldsymbol{u}_{\varepsilon}), \frac{\partial \boldsymbol{F}(\boldsymbol{U} + \boldsymbol{u}_{\varepsilon})}{\partial t}, P + p_{\varepsilon}\right) \tag{5}$$

we can linearise $\boldsymbol{P}$ by expanding about the current state $\boldsymbol{U}$ using the directional derivative

$$P_{ij}(\boldsymbol{U}+\boldsymbol{u}_{\varepsilon}) = P_{ij}(\boldsymbol{U}) + D[\underbrace{P_{ij}(\boldsymbol{F})}_{①}][\boldsymbol{u}_{\varepsilon}] + D\left[\underbrace{P_{ij}\left(\frac{\partial \boldsymbol{F}}{\partial t}\right)}_{②}\right][\boldsymbol{u}_{\varepsilon}] + D[\underbrace{P_{ij}(P)}_{③}][p_{\varepsilon}] + \mathcal{O}(\varepsilon^2) \tag{6}$$

Using the chain rule, ① can be expressed as

$$D[P_{ij}(\boldsymbol{F})][\boldsymbol{u}_{\varepsilon}] = \frac{\partial P_{ij}}{\partial F_{mn}} : D[F_{mn}][\boldsymbol{u}_{\varepsilon}] \tag{7}$$

The directional derivative on the right-hand side of Eq. 7 can then be written as follows:

$$D[F_{mn}][\boldsymbol{u}_{\varepsilon}] = \lim_{\varepsilon \to 0} \frac{F(\boldsymbol{U} + \varepsilon \boldsymbol{u}_{\varepsilon})_{mn} - F(\boldsymbol{U})_{mn}}{\varepsilon} =$$

$$= \lim_{\varepsilon \to 0} \frac{(\nabla_{\boldsymbol{X}}(\boldsymbol{U} + \varepsilon \boldsymbol{u}_\varepsilon) + \mathbb{1})_{mn} - (\nabla_{\boldsymbol{X}}\boldsymbol{U} + \mathbb{1})_{mn}}{\varepsilon} =$$

$$= \lim_{\varepsilon \to 0} \frac{\nabla_{\boldsymbol{X}}(\varepsilon \boldsymbol{u}_\varepsilon)_{mn}}{\varepsilon} \underset{\text{L'Hôpital rule}}{=} (\nabla_{\boldsymbol{X}}\boldsymbol{u}_\varepsilon)_{mn} \tag{8}$$

In the same way, the chain rule applies to ② in Eq. 6, giving

$$D\left[P_{ij}\left(\frac{\partial \boldsymbol{F}}{\partial t}\right)\right][\boldsymbol{u}_\varepsilon] = \frac{\partial P_{ij}}{\partial\left(\frac{\partial F_{mn}}{\partial t}\right)} : D\left[\frac{\partial F_{mn}}{\partial t}\right][\boldsymbol{u}_\varepsilon] \tag{9}$$

where

$$D\left[\frac{\partial F_{mn}}{\partial t}\right][\boldsymbol{u}_\varepsilon] = \lim_{\varepsilon \to 0}\left(\frac{\partial F(\boldsymbol{U} + \varepsilon \boldsymbol{u}_\varepsilon)_{mn}}{\partial t} - \frac{\partial F(\boldsymbol{U})_{mn}}{\partial t}\right)\Big/\varepsilon =$$

$$= \lim_{\varepsilon \to 0}\left(\frac{\partial(\nabla_{\boldsymbol{X}}(\boldsymbol{U} + \varepsilon \boldsymbol{u}_\varepsilon) + \mathbb{1})_{mn}}{\partial t} - \frac{\partial(\nabla_{\boldsymbol{X}}\boldsymbol{U} + \mathbb{1})_{mn}}{\partial t}\right)\Big/\varepsilon =$$

$$= \lim_{\varepsilon \to 0}\left(\frac{\partial \nabla_{\boldsymbol{X}}(\varepsilon \boldsymbol{u}_\varepsilon)_{mn}}{\partial t}\right)\Big/\varepsilon \underset{\text{L'Hôpital rule}}{=} \left(\nabla_{\boldsymbol{X}}\frac{\partial \boldsymbol{u}_\varepsilon}{\partial t}\right)_{mn} \tag{10}$$

Lastly, we can express ③ as

$$D[P_{ij}(P)][p_\varepsilon] = \frac{\partial P_{ij}}{\partial P} : D[P][p_\varepsilon] \tag{11}$$

where the directional derivative on the right-hand side of Eq. 11 can be rewritten as

$$D[P][\boldsymbol{X}] = \lim_{\varepsilon \to 0} \frac{P(\boldsymbol{X} + \varepsilon \boldsymbol{X}) - P(\boldsymbol{X})}{\varepsilon} =$$

$$= \lim_{\varepsilon \to 0} \frac{P + \varepsilon p_\varepsilon - P}{\varepsilon} = p_\varepsilon \tag{12}$$

Substituting Eq. 8, 10 and 12 in Eq. 7, 9 and 11, respectively, we obtain a new version of ①, ② and ③ which, once replaced in Eq. 6, returns the linearisation of the perturbed version of $\boldsymbol{P}$

$$\boldsymbol{P}(\boldsymbol{U} + \boldsymbol{u}_\varepsilon) \simeq \boldsymbol{P}(\boldsymbol{U}) + \nabla_{\boldsymbol{F}}\boldsymbol{P} : \nabla_{\boldsymbol{X}}\boldsymbol{u}_\varepsilon + \nabla_{\frac{\partial \boldsymbol{F}}{\partial t}}\boldsymbol{P} : \nabla_{\boldsymbol{X}}\frac{\partial \boldsymbol{u}_\varepsilon}{\partial t} + \frac{\partial \boldsymbol{P}}{\partial P}p_\varepsilon \tag{13}$$

Finally, since $\frac{\partial \boldsymbol{P}}{\partial P} = J\boldsymbol{F}^{-T}$, we obtain

$$\boldsymbol{P}(\boldsymbol{U} + \boldsymbol{u}_\varepsilon) \simeq \boldsymbol{P}(\boldsymbol{U}) + \nabla_{\boldsymbol{F}}\boldsymbol{P} : \nabla_{\boldsymbol{X}}\boldsymbol{u}_\varepsilon + \nabla_{\frac{\partial \boldsymbol{F}}{\partial t}}\boldsymbol{P} : \nabla_{\boldsymbol{X}}\frac{\partial \boldsymbol{u}_\varepsilon}{\partial t} + p_\varepsilon J\boldsymbol{F}^{-T} \tag{14}$$

The same perturbation analysis can be employed to linearise the Jacobian of the perturbed macro-deformation, $J(\boldsymbol{U} + \boldsymbol{u}_{\boldsymbol{\varepsilon}})$, using [2]. Again, Taylor's expansion of J around the macro-deformation $\boldsymbol{U}$ is written as

$$J(\boldsymbol{U} + \boldsymbol{u}_{\boldsymbol{\varepsilon}}) = J(\boldsymbol{U}) + D[J][\boldsymbol{u}_\varepsilon] + \mathcal{O}(\varepsilon^2) \tag{15}$$

Using the chain rule and remembering Eq. 8, the directional derivative in Eq. 15 is expressed as

$$D[J][\boldsymbol{u}_\varepsilon] = \nabla_{\boldsymbol{F}}J : D[\boldsymbol{F}][\boldsymbol{u}_\varepsilon]$$

$$= \nabla_{\boldsymbol{F}}J : \nabla_{\boldsymbol{X}}\boldsymbol{u}_\varepsilon \tag{16}$$

Replacing Eq. 16 in Eq. 15 and knowing that $\nabla_{\boldsymbol{F}}J = J\boldsymbol{F}^{-T}$, it follows that

$$J(\boldsymbol{U} + \boldsymbol{u}_{\boldsymbol{\varepsilon}}) \simeq J(\boldsymbol{U}) + J\boldsymbol{F}^{-T} : \nabla_{\boldsymbol{X}}\boldsymbol{u}_\varepsilon \simeq$$

$$\simeq J(\boldsymbol{U}) + JF^{-1}_{ji}\frac{\partial (u_\varepsilon)_j}{\partial X_i} \simeq$$

$$\simeq J(\boldsymbol{U}) + J\frac{\partial X_i}{\partial x_j}\frac{\partial (u_\varepsilon)_j}{\partial X_i} \simeq J(\boldsymbol{U}) + J\frac{\partial (u_\varepsilon)_j}{\partial x_j} \tag{17}$$

or, more compactly

$$J(\boldsymbol{U}+\boldsymbol{u_\varepsilon}) \simeq J(\boldsymbol{U})\left(1+\nabla_{\boldsymbol{x}}\cdot\boldsymbol{u}_\varepsilon\right) \tag{18}$$

Since the mass must be conserved at all times, both $J(\boldsymbol{U}+\boldsymbol{u_\varepsilon})-1=0$ and $J(\boldsymbol{U})-1=0$ are valid. It then follows that

$$\begin{aligned} J(\boldsymbol{U}+\boldsymbol{u}_\varepsilon)-1 &\simeq J(\boldsymbol{U}) + J(\boldsymbol{u})\nabla_{\boldsymbol{x}}\cdot\boldsymbol{U}_\varepsilon - 1 \\ &\simeq (J(\boldsymbol{U})-1) + J(\boldsymbol{U})\nabla_{\boldsymbol{x}}\cdot\boldsymbol{u}_\varepsilon \\ &\simeq J(\boldsymbol{U})\nabla_{\boldsymbol{x}}\cdot\boldsymbol{u}_\varepsilon = 0 \end{aligned} \tag{19}$$

As a consequence, $\nabla_{\boldsymbol{x}}\cdot\boldsymbol{u}_\varepsilon = 0$. Replacing this in Eq. 18, we obtain the linearisation of the Jacobian of the perturbed macro-deformation:

$$J(\boldsymbol{U}+\boldsymbol{u}_\varepsilon) \simeq J(\boldsymbol{U}) \tag{20}$$

To summarise, the linearised form of $\boldsymbol{P}^\varepsilon$ and of J^ε, here reported, reads

$$\boldsymbol{P}^\varepsilon \simeq \boldsymbol{P} + \nabla_{\boldsymbol{F}}\boldsymbol{P} : \nabla_{\boldsymbol{X}}\boldsymbol{u}_\varepsilon + \nabla_{\frac{\partial \boldsymbol{F}}{\partial t}}\boldsymbol{P} : \nabla_{\boldsymbol{X}}\frac{\partial \boldsymbol{u}_\varepsilon}{\partial t} + p_\varepsilon J\boldsymbol{F}^{-T} \tag{21a}$$

$$J^\varepsilon \simeq J. \tag{21b}$$

By replacing Eq. 21a and Eq. 21b into the perturbed equations 4a and 4b, and after re-casting the spatial derivatives from the reference into the current configuration, the modified set of equations of motion for a viscoelastic material can be written as

$$\rho\frac{\partial^2\boldsymbol{u}_\varepsilon}{\partial t^2} - \nabla_{\boldsymbol{x}}\cdot\left(\boldsymbol{\mathcal{C}} : \nabla_{\boldsymbol{x}}\boldsymbol{u}_\varepsilon + \boldsymbol{\mathcal{V}} : \nabla_{\boldsymbol{x}}\frac{\partial \boldsymbol{u}_\varepsilon}{\partial t} + p_\varepsilon\mathbb{1}\right) = 0 \qquad \Omega \tag{22a}$$

$$\nabla_{\boldsymbol{x}}\cdot\boldsymbol{u}_\varepsilon = 0 \qquad \Omega \tag{22b}$$

where the fourth-order elasticity and viscosity tensors, $\boldsymbol{\mathcal{C}}$ and $\boldsymbol{\mathcal{V}}$, are functions of the deformation field and of the applied stress:

$$\boldsymbol{\mathcal{C}} = \frac{1}{J}\nabla_{\boldsymbol{F}}\boldsymbol{P}\boldsymbol{F}^T\boldsymbol{F}^T \qquad \text{and} \qquad \boldsymbol{\mathcal{V}} = \frac{1}{J}\nabla_{\frac{\partial \boldsymbol{F}}{\partial t}}\boldsymbol{P}\boldsymbol{F}^T\boldsymbol{F}^T. \tag{23}$$

Substituting the periodic motion defined in Eq. 3, the following set of equations is obtained:

$$\rho\omega^2\boldsymbol{u}_\mathbb{C} + \nabla_{\boldsymbol{x}}\cdot\left((\boldsymbol{\mathcal{G}}' + i\boldsymbol{\mathcal{G}}'') : \nabla_{\boldsymbol{x}}\boldsymbol{u}_\mathbb{C} + p_\mathbb{C}\mathbb{1}\right) = 0 \tag{24a}$$

$$\nabla_{\boldsymbol{x}}\cdot\boldsymbol{u}_\mathbb{C} = 0 \tag{24b}$$

where Eq. 24b indicates the incompressibility of the material subjected to the micro-deformation $\boldsymbol{u}_\mathbb{C}$, while Eq. 24a determines the dynamic behaviour of the propagating shear waves through the real and imaginary components of the complex viscoelasticity tensor, $\boldsymbol{\mathcal{G}}^* = \boldsymbol{\mathcal{G}}' + i\boldsymbol{\mathcal{G}}''$.

References

1. Capilnasiu A, Hadjicharalambous M, Fovargue D, Patel D, Holub O, Bilston L, et al. Magnetic resonance elastography in nonlinear viscoelastic materials under load. Biomech Model Mechanobiol. 2019;18(1):111–135. doi:10.1007/s10237-018-1072-1.

2. Bonet J, Wood RD. Nonlinear continuum mechanics for finite element analysis. Cambridge University Press; 1997. Available from: https://books.google.co.uk/books?hl=en{&}lr={&}id=ORmLdrq1fI8C{&}oi=fnd{&}pg=PR13{&}dq=Bonet+Wood{&}ots=9NUkcdEDer{&}sig=bxdccXJ3j61XuTKz5Qm45edLFxE{#}v=onepage{&}q=BonetWood{&}f=false.
