# Supplementary material for: Impact of axisymmetric deformation on MR elastography of a nonlinear tissue-mimicking material and implications in peri-tumour stiffness quantification
Source: PLoS One. 2021 Jul 9;16(7):e0253804. doi: 10.1371/journal.pone.0253804 (PMC8270167; doi:10.1371/journal.pone.0253804)
Supplement: Attachment — Submitted filename: Response to Reviewers.pdf [file pone.0253804.s002.pdf]

Marco Fiorito, PhD
University of Basel, AMT Centre, Department of Biomedical Engineering
Gewerbestrasse 14, 4123 Allschwil

Allschwil, 18th April 2021

Emily Chenette, PhD
Deputy Editor-in-Chief
PLOS One

Dear Dr. Chenette,

We would like to thank the reviewer for the constructive suggestions and feedback which we found valuable to improving our manuscript. We have addressed the comments in full in the responses following this letter, with reference to the revised sections of the manuscript. For clarity, the modified parts have been written in red. Please find below a point-by-point response to each comment.

Yours sincerely,

Marco Fiorito

1. I would recommend to review the title, authors present a general formulation of finite strains and apply it to determine the mechanical state of a non-living material in which a defect with the shape of a sphere is introduced and them a pressure inserted. There are few reference to the tumour in the main body of the text.

Following your suggestion, we have revised the title to make clear that a phantom setup is used. In addition, as described in our response to question 11, the relevance of the work to tumours have been clarified throughout the text.

While the mechanical state of a tumour is undeniably more complex, we believe the work presented is a useful model for dealing with nonlinearities in MRE quantification and represents a starting point to understand the induced solid stress for clinical assessment of tumour progression. For these reasons, we believe that the reference to the tumour should be kept in the title and the main body of the text, subject to clear indication of the assumptions.

2. The authors perform a theoretical study which is far from mimic the real environment of a growing tumour, I would recommend to rephrase or remove the sentences related to the early application of the model to predict the mechanical state of a real tumour (for exaple the lass sentence of the abstract "a significant step …. treatment efficacy". In fact, they do not perform any validation neither quantitative nor qualitative with real tissue data.

As per the suggestion, we have revised the last part of the abstract, highlighting the need to translate these findings into real tissue. The further challenges on the modelling side are also weighed against the current experimental limitations in the last paragraph of the Discussion, from line 565. At line 582, a reference to a promising work by Fovargue *et al.* (2019) highlights the first clinical application of the presented formulation, that combines MRE reconstruction and nonlinear tissue mechanics in order to retrieve the underlying pressure generated by invasive breast tumours. The conclusions were also reformulated to focus on the analytical model, leaving further implications regarding tumour growth to the discussion.

3. The authors present a complete analytical formulation for finite strains which they introduced in a previous work (Capilnasiu et al., 2019). In my opinion the authors should explain the new contributions in further detail and move the sections related to the general formulation already published to supplementary material. In fact, it is hard to see the novelty of this formulation against their previous work and the analytical part related to the sphere could be further elaborated.

We agree that the previous paper structure did not help to emphasise the novel contributions of this work. To account for this we have moved the derivation of the perturbed version of the equations of motion, previously introduced by Capilnasiu *et al.* (2019) to the appendix as suggested. The section "Linearised Constitutive Laws of a Large-Strain Viscoelastic Body Subjected to Small Perturbations" was then revised, starting from line 91 to line 107, and only the essential parts required for the development of the thick-shelled sphere approximation were kept.

Moreover, to highlight the differences between our manuscript and the paper published by Capilnasiu *et al.* (2019), we further clarified that the latter focuses around the impact of uniaxial compression on

the MRE reconstruction of the shear modulus for a nonlinear viscoelastic material. The present work used Capilnasiu's framework but expanded their formulation to the case of a spherical inflation, which is qualitatively closer and relevant to a tumour geometry. Changes were therefore applied at line 43, 66 and 81. Further references to Capilnasiu's paper are presence at line 564.

4. Lines 14-15. The authors assume the tumour is a perfect sphere. This is a rather crude assumption in a real tumour growing in a very heterogeneous environment in the human body please at least state the conditions to consider the tumour a perfect sphere.

Please refer to our response to question 11.

5. The use of the name "a" for different variables is confusing (internal spatial radius page 5 and 6, subscript equation (21), and shear deformation line 158) please rename the different variables with different names.

We kept the name "a" for the internal spatial radius at page 4 and 5.

We have replaced it with "l" in the subscripts in Eq. 11a,b,c.

We have replaced it with "β" in Eq. 13 and Eq. 22.

6. Line 140. The authors are assuming the "sigma_rr" in the external radius (r=b) is zero, however it is surrounded by additional tissue, please, justify this assumption.

As explained in the paragraph in line 108, the two radii r=a and r=b denote the inner and outer boundaries of the thick shelled sphere in the inflated state. The outer radius may be considered as e.g. the boundary of the organ containing the tumour. In our formulation, we have assumed that if r=b is sufficiently distant from r=a, then we may consider that the radial stress σ_{rr} at position r=b has reached 0 kPa. Although these assumptions can be adjusted in specific scenarios – i.e. a different pressure could be associated to location r=b – in the absence of data, we have chosen set this boundary condition to 0 kPa. Nonetheless, the presented model can be adapted in the case of additional information regarding the state of stress of the tissue around the tumour, which would lead to a somewhat different evaluation for p_i (Eq. 10). This does not fundamentally change the approach we presented. To make these points clearer, we have added an explanation at line 120.

7. Table 1, lines 196-206. Even though, it is stated in the main text (line 205), I think it would be good to include also in the table legend if the percentage of compression in the micro scale refer to a percentage of the macro scale deformation 13% or the total deformation of the sample.

We agree that Table 1 could be confusing to readers and have revised the headings for clarity. Moreover, in the caption we have explicitly described the difference between the applied harmonic micro-/macro-compression and the uniaxial compression.

8. Figure 1, it is the same as figure 2 in Capilnasiu et al. (2019), Just the number change, please try to change the figure a little bit to make it clearer.

The experiments were performed using the same instrumentation and a standard protocol as those used by Capilnasiu *et al.* (2019). Although the resulting figures may appear similar superficially, we believe that this is the optimal way to present the results as long as the numbers involved in these particular experiments are unambiguous. It is not clear to us how this figure should be modified as we do not believe it was unclear in the original state.

9. Lines 298-299. "the non-spherical shape of the balloon when not fully inflated". Please, specify the moment from which the balloon could be considered inflated, and the implications in your formulation since you are considering a perfect sphere.

Please refer to our response to question 11.

10. Lines 311-313. You introduce a complete viscoelastic model however them you just use the elastic part. This is really confusing, please disscuss in the Discussion Section.

In brief, because the viscous dominance in the material was small (as shown by the small fractional derivative order α), we were able to use the simplifying assumption outlined in line 217 where the viscous part of the model is more easily accounted for, and the form of mu is simpler (Eq. 23). Accordingly, our experimental set up was designed to measure pressure at steady state – this combined with Eq. 23 allowed the determination of the material parameters.

Indeed, the general form of the equations of motion, which represent the starting point for the development of our model, have been derived using a complete viscoelastic formulation. This led to the definition of two tensors: **G'** and **G"**, defined in Eq. 4. In the section titled "Wave Propagation in Expanded Thick-Shelled Sphere", a purely elastic material simplification was made, and Eq. 9 contained no viscous term. This assumption did not only provide a better representation of the experimental conditions, but also made the model more manageable. While this was briefly explained in line 279 of the Materials & Methods, we agree that a justification of the reasons that led us to focus on a purely elastic material law would be useful in the section where the model is presented. We have thus provided a new explanation at line 126 and reinforced these concepts in the Discussion at line 481. Finally, the mild viscosity (small α, see Eq. 20) observed through the rheologic characterisation of the soft plastic material supports the purely elastic assumption, as described at lines 548-559.

11. Figure 3. The dimensions of the inclusion sphere compare to the dimensions of the paths for catheter and other devices inclusions are of the same order of magnitude. Please, comment on that and the consequences in the formulation.

For this paper we have chosen to provide a general model to investigate the mechanical impact of the strain generated by a pressurised tumour onto its surroundings. To do so, we have based our formulation on the assumption that a tumour could be approximated by a sphere. Indeed, this

provides a crude representation of the heterogeneous conditions encountered in the tumour environment. At line 453, we have highlighted how tumour shape can differ from the idealisation presented in this model. Nonetheless, we believe a spherical approximation is a logical starting point when it comes to modelling intra- and peri-tumour mechanics, as it is often beneficial to approach a problem with a simpler model before increasing its complexity. The analytically tractable model we employed was able to capture the key mechanisms we set out to test – and while these results can be extended to the more realistic settings this will likely have to involve numerical simulations, which are far more complex to interpret. The current model provides a baseline against which secondary effects of geometrical variation can be benchmarked.

Incidentally, our experimental tumour phantom geometry featured protrusions and did not remain an ideal sphere throughout the experiments, though neither of these were intentional. For the validation of the theory, a balloon catheter inserted inside a viscoelastic cuboid was used to mimic the stress of a pressurised tumour onto the host tissue. While this set up generated an axisymmetric inflation, a sphere was only closely achieved when the maximal volume of water (0.4 mL) was injected. This is caused by the balloon being located some distance back along the catheter shaft and not at the tip (Fig 3) and could be assessed by the analysis of the segmented MR images of the balloon. We have added a table (Table 2) to report the values of principal axis at various states of inflation. While one of the principal axes is aligned and fixed with the catheter line, the other axes grew in relative magnitude at each inflation state. The ratio between the three axis reached values close to 1 only at the last inflation state. These points were clarified in the method section, at line 284. Because of the particular manner in which the balloon underwent inflation (whereby the equatorial region would bulge out before the poles) the quoted major axis lengths are maximal (worst-case) estimates making the balloon more spherical in real life – however, the precise shape could not be determined due to imaging resolution limit.

Additional complications were encountered due to the presence of the catheter access hole created to place the balloon at the desired depth into the plastisol cuboid. The segmented catheter line is on a comparable order of magnitude to the balloon at low inflation states and could have impacted the MRE reconstruction, and the hole itself could affect the mechanical properties of the material. Curing the plastisol with the catheter already in place would have been the preferred solution, but could not be done due to the high temperatures involved. In order to minimise these issues, MRE data were acquired in the imaging plane orthogonal to the direction of the catheter line near the equator where the balloon geometry most closely resembled a sphere. The catheter line was then absent in all central slices. This way, the mechanical effects were sampled from selected regions where the confounding effects are minimal, at the same time avoiding the resolution limit at the polar region that increasingly hamper the analysis. The mechanical effects evaluated are still fully 3D and anisotropic in nature – we simply looked for these where the signal-to-noise (or effect-to-noise) is expected to be maximum. Ultimately, we believe the level of agreement between the modelled shear strain and the probed stretch index demonstrates the effectiveness of these mitigating measures. These measures have been newly described at line 329 and discussed from line 464.

12. In the conclusion authors state that “tumour- generated stress has been investigated” however in the discussion section there is just a brief discussion of two previous work related to tumour-generated stress. Authors centre the discussion on the validation of the analytical model with their experiments on plastisol. Authors should further discuss on the limitations on

their model to extrapolate results to real tumours and the advance of his work against what has already been published in tumour growth.

We thank the reviewer for this comment. To address this point, we have reformulated the last part of the Discussion. Starting from line 565, we clarified the work left to be done to advance this model to account for real tumours, as well as parallel developments which incorporated the ideas in the present model into MRI reconstruction to retrieve the pressure generated by an idealised tumour. We further note that a preliminary clinical application of this method has been reported on breast tumour images and is currently undergoing further study. We believe that these descriptions will help to set up the broader context in which our work sits, and the illustrate its potential for translation into a clinical scenario. Nonetheless, many of the limitations encountered in the MRE reconstruction (e.g.: discontinuity between plastisol and balloon; the water inside the balloon does not support shear waves; the presence of the catheter line) will be alleviated.
